# Draft Genome Assembly of an Iconic Arctic Species: Muskox (*Ovibos moschatus*)

**DOI:** 10.3390/genes13050809

**Published:** 2022-05-01

**Authors:** Erin Prewer, Susan Kutz, Lisa-Marie Leclerc, Christopher J. Kyle

**Affiliations:** 1 Environmental and Life Sciences Graduate Program, Trent University, 1600 West Bank Drive, Environmental Science Building, Peterborough, ON K9J 7B8, Canada; christopherkyle@trentu.ca; 2 Department of Ecosystem and Public Health, Faculty of Veterinary Medicine, University of Calgary, 3280 Hospital Drive NW, Calgary, AB T2N 4Z6, Canada; skutz@ucalgary.ca; 3 Department of Environment, Government of Nunavut, P.O. Box 377, Kugluktuk, NU X0B 0E0, Canada; lleclerc@gov.nu.ca; 4 Forensic Science Department, Trent University, 1600 West Bank Drive, DNA Building Block B, Peterborough, ON K9L OG2, Canada; 5 Natural Resources DNA Profiling and Forensic Centre, 2140 East Bank Drive, Peterborough, ON K9L 1Z8, Canada

**Keywords:** de novo genome assembly, genetically depauperate, muskoxen, Arctic, *Ovibos moshcatus*, PSMC, phylogenetic tree

## Abstract

Muskoxen (*Ovibos moschatus*) are Arctic species within the Caprinae subfamily that are economically and culturally significant to northern Indigenous communities. Low genetic diversity from repeated genetic bottlenecks, coupled with the effects of Arctic warming (e.g., heat stress, changing forage, pathogen range expansions), present conservation concerns for this species. Reference genome assemblies enhance our ecological and evolutionary understanding of species (which in turn aid conservation efforts). Herein, we provide a full draft reference genome of muskox using Illumina Hiseq data and cross-species scaffolding. The final reference assembly yielded a genome of 2,621,890,883 bp in length, a scaffold N50 of ~13.2 million, and an annotation identifying ~19.3 k genes. The muskox genome assembly and annotation were then used to reconstruct a phylogenetic tree which estimated muskoxen diverged from other ungulate species~12 Mya. To gain insight into the demographic history of muskoxen we also performed pairwise sequentially Markovian coalescent (PSMC) that identified two population bottlenecks coinciding with major glaciation events contributing to the notoriously low genetic variation observed in muskoxen. Overall, this genome assembly provides a foundation for future population genomic studies, such as latitudinal analyses, to explore the capacity of muskoxen to adapt to rapidly changing environments.

## 1. Introduction

Muskoxen (*Ovibos moschatus*) are an iconic Arctic species closely related to sheep and goats within the *Caprinae* subfamily and the only living member of the *Ovibos* genus. Endemic muskoxen are found on the mainland and Arctic Archipelago of the Northwest Territories (NWT) and Nunavut (NU), Canada and east Greenland (Denmark). Reintroduced or translocated populations of muskoxen are currently found in west Greenland, Russia, Alaska (USA), and portions of the Yukon and Quebec (Canada) [1]. Muskoxen are keystone Arctic species, facilitating nitrogen and soil nutrient turnover while also having significant cultural, nutritional, and economic roles for Indigenous People of the Arctic [2,3]. Muskoxen contribute to community identity in the creation of art, tools, and clothing, but also local food security, employment, and revenue through sport hunting and sales of muskox by-products such as its specialized wool, qiviut [3]. Harvests were suspended in 2012 due drastic declines in muskox populations on Banks Island (NWT) and Victoria Island (NU) [4,5]. These population declines occurred concurrently with changing climatic conditions including severe icing events, vegetation shifts, and northward range expansions of pathogens [1,6,7,8]. As Banks and Victoria Island populations were two of the largest endemic populations, these declines incited concern for muskox health and sustainability [1,4].

Muskox populations have notoriously low levels of genetic diversity resulting from a combination of population bottlenecks, founder effects, and population fragmentation across their range [9,10,11,12,13]. Confounding the premise that low genetic diversity may be associated with population declines on Victoria and Banks Island is the fact that other populations across Canada remained stable or grew with unknown reasons for this dichotomy [1]. Past diversity estimates from microsatellite studies [9,12,13], as well as genotyping by sequencing by Hansen et al. [14], focused on neutral regions of the genome. While informative, these studies did not elucidate information related to the adaptive capacity of muskoxen that might be associated with demographic trends in context of changing selective factors on the landscape (such as those from climate change and a warming Arctic). Elucidating the functional genomic variation across muskox populations may provide insight into muskox population health and vulnerabilities but requires a better genomic foundation. Access to a reference genome would offer the ability to assess the genetic diversity of genes linked to evolutionary and local muskox adaptations such as increased digestion, cold resistance, and immune response [15,16,17]. As such, genome assemblies have the potential to provide insight into how evolutionary and demographic histories have influenced patterns of genomic diversity in muskoxen as well as their capacity to adapt to a rapidly changing environment.

Herein, we report the first assembly and annotation of the muskox genome using high throughput Illumina sequencing and cross-species in silico mate pair library construction. Our final genome assembly and annotation was used to reconstruct a phylogenetic tree and estimate divergence times of this unique genus from other ungulate species. The genome was also used to perform pairwise sequentially Markovian coalescent (PSMC) analyses to identify historical trends in an effective population size. Our main goal was to provide a reference genome from which further population genomic studies could be performed, such as assessing the distribution of genetic diversity in muskoxen, identifying genes involved in their unique Arctic adaptations, and to better understand their capacity to respond to rapid environmental change.

## 2. Materials and Methods

### 2.1. Genome Sequencing

Genomic DNA for the reference genome assembly was extracted from the hide of a male muskox from Holman, Victoria Island, Canada using the Qiagen DNeasy blood and tissue kit (Qiagen, Mississauga, ON, Canada). Quality of extracted DNA was assessed on a 1.5% agarose gel and quantified using PicoGreen. Extracted DNA was shipped on dry ice to the Centre for Applied Genomics at Sick Kids hospital, Toronto, ON, CA for library preparations and sequencing. Four paired-end libraries were prepared using Illumina TruSeq Nano DNA kit (Illumina, San Diego, CA, USA) with inserts sizes of 200 bp, 350 bp, 550 bp and 700 bp and two mate-pair libraries were created using the NxSeq Long Mate Pair Library Kit with insert sizes of 5 kbp and 8 kbp. Two paired-end libraries, with insert sizes 200 bp and 350 bp, were sequenced on the HiSeqX which produced approximately 940 million (2 × 151 bp) paired reads. The two additional, paired-end libraries with insert sizes of 550 bp and 700 bp, as well as two mate-pair libraries, were sequenced on the HiSeq2500 producing approximately 237 million (2 × 126 bp) paired reads.

### 2.2. De Novo Genome Assembly

We performed a series of data filtering steps to remove read contamination, low quality reads, and duplicate reads. We used FastQC v0.11.5 [18] to check overall quality of the libraries after each step to assess how much data was removed and effectiveness of each program. First, we removed adapter and primer sequence contamination using scythe v0.994 [19] and EA utils v. 805 [20], with mate-pair libraries undergoing additional processing to split and remove chimera code, junction code and linker sequences as per the Lucigen NxSeq long mate-pair library kit protocol. Next, we performed low quality base trimming using sickle v. 1.33 [21] with a quality score cut off set at Q = 25 and a minimum length set at 70% of the original read length. We then removed duplicate read pairs using FastUniq v. 1.1 [22] in order to remove any technical duplicates which may affect downstream scaffolding. Finally, we used bbsplit from the bbmap v37.22 [23] package to remove sequences belonging to known lab contaminants using viral, bacterial, fungal and human database created by DeconSeq [24]. After data preprocessing, approximately 1.23 × 10^11^ bp remained from paired-end libraries and approximately 4.93 × 10^6^ bp remained from mate-pair libraries for a theoretical coverage of ~85× based on the genome size of the domestic goat (2.9 Gb).

We performed an initial de novo assembly using SOAPdenovo2 v.240 software [25], that is, a De Bruijn graph-based de novo assembler and then used Gapcloser v.1.12-r6 [25] and uses short read library data to fill gaps that occur during scaffolding. To further improve the de novo genome assembly, we used cross-species-scaffolding v 1.0.1 [26]. This reference-based scaffolding method uses genomes of closely related species to create scaffolding libraries in silico that can then be used to re-perform scaffolding on assembled genomes. Cross-species-scaffolding requires a closely related genome, so we tested both domestic goat (GCA_001704415.1) and domestic sheep genomes (GCA_002742125.1) to create two separate sets of 20 mate-pair libraries with inserts ranging from 500 bp to 50 kbp. To determine which in silico library (goat or sheep based) produced the best overall assembly we used both datasets to scaffold and assemble multiple genomes with SOAPdenovo2, with kmers ranging from 51 to 127 bp. These genomes were then ranked using metrics estimated by Quast v4.4 [27] and BUSCOv3 [28], including NG50, number of Ns per 100 kbp and completed BUSCOs (Supplementary Table S2) to identify which kmer value, and reference-based mate pair library, was optimal for downstream analyses.

Large sections of the muskox’s mitochondrial genome were found within several long scaffolds of the top assembly. To address this issue, we further filtered cleaned paired-end and mate-pair reads to remove reads that corresponded to the mitochondrial genome. Mitochondrial reads were extracted from cleaned sequence libraries using the bbsplit tool from bbmap by binning reads that mapped to the reference mitochondrial genome (GenBank FJ207536.1). Then, to create the mitochondrial genome, these reads were aligned to the reference (FJ207536.1) and the vcfutils.pl tool from the samtools package was used to call consensus sequences using default parameters. Finally, we assembled a new whole nuclear genome using SOAPdenovo2 with the newly filtered clean read dataset. This genome assembly was then used to re-perform genome scaffolding with the previously optimized cross-species-scaffolding library and kmer value. The final genome assembly was completed by a final round of Gapcloser.

### 2.3. Genome Annotation

We used Repeat Modeler v.1.0.10 [29] software to create repeat libraries for the muskox genome assembly. We then used these libraries to perform hard masking of repetitive regions in the muskox genome using Repeat Masker v. 4.0.7 software [30]. We retrained Augustus v. 3.2.3 software [31] to perform gene prediction on the masked genome. In order to create a set of training genes for Augustus, we used Gmap v2017-08-15 [32], Genemark v4.35 [33], Exonerate v. 2.2.0 [34] and EVidenceModeler v.1.1.1 software [35]. Genemark-ES was used to perform ab initio gene prediction on both masked genomes. We downloaded all sheep and goat EST sequences from GenBank and used them to perform evidence-based gene prediction on the masked genomes using Gmap software. We also downloaded all sheep and goat SWISS-PROT reviewed protein sequences from the UniProt-Kb database and performed evidence-based gene prediction of masked genomes using exonerate software. We used EVidenceModeler v.1.1.1 software to create a set of consensus genes from outputs produced by Genemark v4.35, Exonerate v. 2.2.0 and Gmap v2017-08-15 software [32,33,34,35] with more weight given to the evidence-based predictions. This set of consensus genes was used to retrain Augustus software, creating a new set of parameters for muskoxen. These parameters were used to perform gene prediction on the masked genomes with the consensus gene set used as hints.

### 2.4. Evolutionary and Demographic History

Pairwise Sequentially Markovian Coalescent (PSMC) model was used to estimate effective population history following the pipeline outlined by Li et al. [36]. Sequences from cleaned paired-end libraries were aligned to the muskox genome using Burrows-Wheeler alignments (BWA) v. 0.7.17 [37]. BAM alignments from each library were merged and duplicates were removed using MergeBamAlignment and MarkDuplicates from the Picard Toolkit (Broad Institute). The combined alignment was used to create consensus sequences using samtools v.0.1.15, vcfutils and bcftools [38]. Option -d was set to 20, as recommended to be set to a third of the coverage, while -D was set at 170, as recommended to be twice the average coverage. We then performed PSMC v.0.6.5 analyses using -p parameter suitable for modern humans (4 + 25*2 + 4 + 6) and bootstrapped using 100 iterations. The mutation rate 2 × 10^−9^ was chosen as the average mutation rate in mammals with a generation time of 10 years [39].

To construct the phylogenetic tree, 1:1 orthologous genes were identified among 8 species (muskox-*Ovibos moschatus*, sheep-*Ovis aries*; GCA_000298735.1, goat-*Capra hircus*; GCA_001704415.1, cow–*Bos taurus*; GCA_002263795.2, horse -Equus caballus; GCA_002863925.1, pig–*Sus scrofa*; GCA_000003025.6, bison–*Bison bison bison*; GCA_000754665.1, yak–*Bos gruniens*; GCA_000298355.1). Using PorthoMCL [40], 1:1 orthologous genes were then identified and corresponding coding sequences were aligned using Clustal-Omega. All alignments were concatenated using FasConCat v.1.04 software [41], and Jmodeltest v.2.1.10 was used to predict the best-fit model of nucleotide substitution [42]. A maximum likelihood tree was then generated in RAxMLv8 [43] using the GTR+I+G model with 1000 bootstrap replicates and horse as the outgroup. MCMCtree from the PAMLv4 package [44] was used to convert the tree to an ultrametric format and to calibrate the nodes with known split times. Calibration minimums and maximums were given for sheep-goat divergence (3.9–8.1 MYA) from Chen et al. [45] and for sheep-cow divergence (18.3–28.5 MYA) from Benton and Donoghue [46].

## 3. Results

### 3.1. Genome Sequencing and Assembly

We sequenced the draft genome of a male muskox from Victoria Island using 4 paired-end libraries and 2 mate-pair libraries. Across all 6 libraries, a total of 1,178,984,124 paired reads were generated. After filtering for low quality, duplicate and contaminant reads, 78.9% (843,572,921) paired reads remained for paired-end libraries, however only 0.04% (39,179) paired reads remained for mate-pair libraries (Supplementary Table S1). Loss of mate pair library data was the result of duplicate read removal steps. Mate pair sequencing failure likely relates to a lack of sufficient quality DNA for mate pair library preparations, and thus PCR bias when sequencing. The overall result was a sequencing coverage of ~85× for the muskox genome based on a goat genome size of 2.9 Gb.

An initial draft assembly was performed by SOAPdenovo2 software v.2.04–r240 [25] using all paired-end libraries and low coverage of mate pair reads that remained after filtering. The resulting genome was fragmented with over ~822 K contigs and an N50 of 26,107 bp, meaning that half of the genome was made up of contigs of this length or larger, and a complete BUSCO score of 65.6% which is below that of other ungulate genomes [45]. We assumed the lower genome quality as reflected by the metrics of # of contigs, N50 and BUSCO scores was likely associated with the lack of mate pair libraries, preventing SOAPdenovo2 v.240 [25] from combining contigs, especially those separated by large gaps or repetitive regions, into scaffolds. In order to improve genome quality, we used in silico mate-pair libraries created by Cross-Species-Scaffolding (v.2.2) [26] by aligning the cleaned muskox reads to those of closely related species. Both sheep and goat genomes were used as a reference, after which assemblies were compared based on resulting N50, scaffold number and BUSCO scores amongst other common quality metrics (e.g., Ns per 100 kbp) to determine which genome would produce the best in silico mate-pair libraries, (Supplementary Table S2). Cross-species scaffolding libraries created by both reference genomes greatly improved genome quality, but the assembly with the goat genome as reference, using a kmer value of 121, ranked highest. The best ranked genome had 6495 contigs, a complete BUSCO score of 87.5% and an N50 of 1,705,149 bp, which is 65× higher than the assembly without cross-species scaffolding. The use of cross-species scaffolding allowed us to create large scaffolds without further sequencing of long read data, mate pair libraries, and/or sequencing by ligation, but there are potential drawbacks when using related species scaffolding methods. First, cross-species scaffolding is limited by the quality of the initial genome assembly as well as the existence of a high-quality genome from a related species, though the increased availability of genomic resources may allow this method to be more widely used [26,47]. For example, in silico mate pair libraries have been used to improve genomes of fin whales, narwhals, gray’s beaked whales as well as addax (that similarly used the goat genome as a reference) [48,49,50,51] Additionally, as the arrangement of the contigs within the scaffolds are based on the genome of another species, this can limit analyses of genomic architecture, such as gene copy number and gene rearrangements [26,47]. Previous karyotype mapping has found muskoxen to be highly homologous to ancestral Pecora chromosomal arrangements, with five fusions of different chromosome arms forming submetacentric chromosomes [52,53,54,55]. However, G−, C− and R− banding found many muskox chromosomes to be either identical or strikingly similar to those of goats. These data further strengthen the validity of using the goat genome as a reference for cross-species scaffolding of muskoxen, and likely explain why we found goats produced a better muskox assembly than the sheep genome [52,53,54,55] The removal of mitochondrial reads did not greatly improve the quality of the initial genome assembly with a complete BUSCO score of 64.2% and an N50 of 26,274 however using this genome as the base to reperform cross-species scaffolding had a large impact on the final genome assembly quality.

The final draft genome produced 8659 scaffolds for a cumulative length of 2,621,890,883 bp, with a contig and scaffold N50 of 38,369 bp and 13,200,690 bp, respectively. The scaffold N50 was 7× better than our previous assembly that used cross-species scaffolding prior to mitochondrial read filtering. The longest scaffold was 50,595,910 bp long, where 2,601,612,364 bp of the assembly was made up of contigs over 25,000 bp in length. Based on cumulative length and Nx plots (Supplementary Figure S1) these quality metrics show that the draft genome was mainly composed of large scaffolds with few short sequences. Completeness of the draft genome was further assessed with Benchmarking Universal Single-Copy Orthologs (BUSCO v.3) [28] vertebrata gene set. Of the 3023 genes in the BUSCO gene set, 2658 (87.9%) complete single copy orthologs were identified in the muskox draft genome, indicating that gene prediction would identify a large percentage of completed genes in muskoxen. Additional BUSCO scores and basic statistics of the assemblies, pre- and post-cross-species scaffolding, as well as pre- and post- mitochondrial read removal, are shown in Table 1. The draft assembly was deposited in GenBank (JACAUE000000000).

### 3.2. Genome Annotation

Repeat masking was performed prior to gene predictions using RepeatMasker (v.4.0.7) [30] that identified 42.39% of sequences were made up of interspersed repeats. Once repeats were masked, both homology and ab initio predictions were used to identify protein coding genes. Genes predicted by both methods were combined using EVidence Modeler (EVM). These genes were then used as a training set for Augustus (v.3.2.3) [31] which performed the final gene prediction with the combined consensus gene set as hints. A total of 19,132 genes were predicted with an average of 24.9 kb per gene, 1461 bp per coding DNA sequence, and 154 bp per exon. Of the predicted genes, 12,848 (67%) were annotated by Interpro for Gene Ontology and 11,349 genes (59%) aligned to the National Centre for Biotechnology Information (NCBI) non-redundant protein database (Supplementary Figure S2). In comparison to the genome annotations of other ungulate species, the number of genes and average coding sequence length of the muskox genome assembly are within the norm [45]. We also compared genomes using quality metrics of contig and scaffold N50s, and complete BUSCO scores. The quality of the draft muskox genome assembled herein falls within those presented by Chen et al. [45] for other ruminant species. Genome assembly and annotation metrics of the final muskox genome are shown in Table 2 in comparison with the highest quality ruminant genomes assembled by Chen et al. [45] as well as their assembly of another Arctic ruminant (reindeer).

### 3.3. Evolutionary and Demographic History

**Figure 1:**
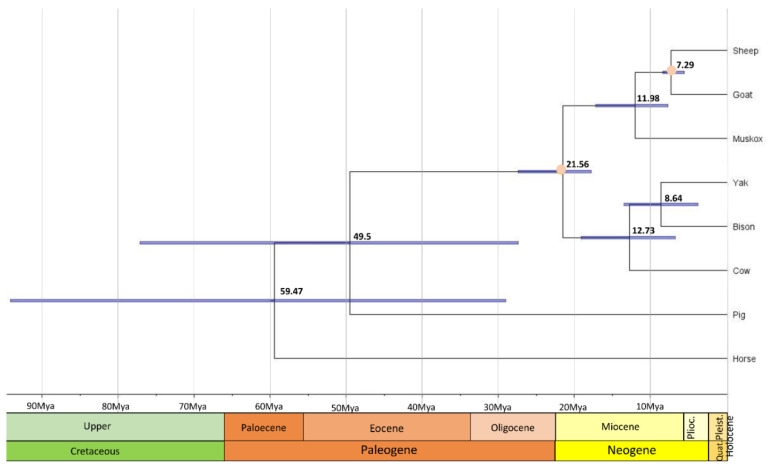
Evolution of gene families among muskoxen and non-Arctic relatives. The phylogenetic tree was constructed using 893 single copy orthologs across all 8 ungulate species. Divergence times are in black, orange dots represent the calibration points, and 95% credible intervals are represented by blue node bars.

In order to compare coding sequences of muskoxen to orthologous genes among Caprinae and other mammals, PorthoMCL was used to identify orthologs amongst 8 ungulate species. The ortholog set was then filtered to contain only 1:1 orthologs and resulting in 893 genes used for the phylogenetic tree. As expected, the ultrametric and time calibrated phylogenetic tree from mcmctree shows that muskoxen are more closely related to sheep and goats than cows (Figure 1) [11,56,57]. From this tree, muskoxen diverged from sheep and goats approximately 12 million years ago (Mya) and this sister clade diverged from cows approximately 21.6 Mya. The divergence time of the sheep and goats’ clade is comparable with times previously estimated in the literature, as are the branch dates of other species included in the tree [45]. Previous studies using the mitochondrial genome estimate muskoxen diverged from sheep and goats ~8–15 Mya [58,59], values that fall within our confidence intervals of 8–17 Mya. Mitochondrial gene sequences have been found to have lower resolving power over nuclear exons when investigating phylogeny reconstructions, therefore the analyses performed based on the nDNA genome assembly should be more accurate [60] in describing the phylogeny and divergence times of this unique genus.

**Figure 2:**
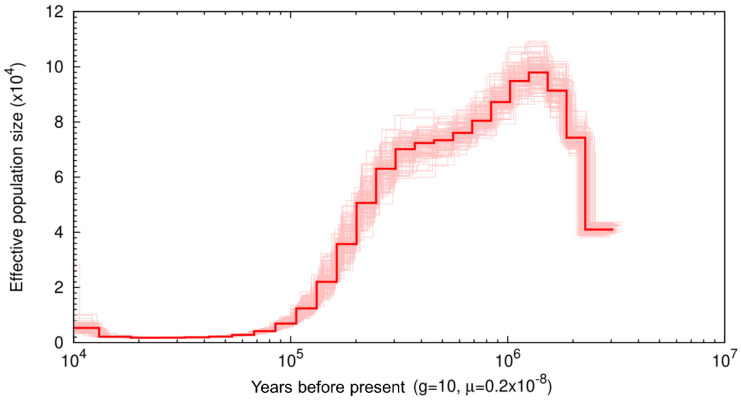
Pairwise sequentially Markovian coalescent (PSMC) plot of the muskox genome with 100 bootstrap repetitions assuming a generation time of 10 and substitution rate of 0.2 × 10^−8^ The *x*-axis represents time before present on a log scale and y axis represents effective population size.

We used the Pairwise Sequentially Markovian Coalescent (PSMC) model to assess historical patterns of effective population size (Ne) (Figure 2) [36]. At 3 Mya, there is a relatively low Ne at ~35,000, and over the next 500 thousand years, Ne increases until it reaches 100 k. At 1 Mya, Ne begins to steadily decline until it reaches an Ne of ~2 k. This low Ne remains from ~20—40 thousand years ago (Kya) before showing a slight recovery ~15 Kya, reaching an Ne ~5 k. Low points in muskox Ne starting at 3 Mya and 40 Kya coincide with major glaciation events. The start of the PSCM occurred during a major glaciation event occurring ~3.15–2.75 Mya, referred to as the climate crash [61] and we see Ne recovery follows immediately after this period. The second low point represents a bottleneck occurring during the last glacial maximum (LGM) ~26.5—19 Kya where once again a recovery is observed only after the LGM ended [62], though the decline of Ne was continuous from 1 Mya onward.

Previous muskox diversity analyses performed by Hansen et al., [14] used ddrad sequencing to assess neutral genome wide variation in muskox populations, where stairway plot analyses were used to assess Ne through time. The analyses by Hansen et al. [14] of the mainland west population, the closest geographical population to the sample used in this study, showed that Ne was at a stable high of ~13 k at 50 Kya until ~27 Kya when Ne began to steadily decline. Hansen et al. [14] showed the population decline started to plateau at ~15 Kya with Ne estimates remaining at ~9 k at 10 Kya. In comparison to the PSMC analyses performed herein, Ne estimates at 10 Kya are nearly doubled in analyses by Hansen et al. [14]. Additionally, where Hansen et al. [14] found population declines occurred between ~27 Kya and ~15 Kya, the PSMC analyses performed in this study found Ne stable or increasing in size between this time frame. Finally, the high Ne reported in Hansen et al. [14] between ~27 Kya and 50 Kya coincides with an Ne low according to PSMC analyses with Ne estimates 6× higher in Hansen et al. [14]. Discrepancies between these two results likely relate to the difference in reference genomes used to call genotypes, where Hansen et al. [14] used the sheep genome, and the PSMC analyses performed herein used the cross-species scaffolded muskox genome. Prasad et al. [47] performed variant calling using the reference genomes of related species of varying phylogenetic distance and compared them to variants called using cross-species scaffolded genomes. When used for downstream analyses such as PSMCs, analyses using cross-species scaffolded genomes were more reliable than mapping directly to the reference genome of related species, even if the cross-species genome was highly fragmented [47]. Overall, Prasad et al. [47] found that if a suitable reference genome did not exist for a species of interest, cross-species scaffolding provides a good reference genome alternative. As such, the use of the muskox genome assembled herein may provide a better reference for demographic analyses such as the PSMCs than published sheep and goat genomes.

## 4. Conclusions

In this study, we present the first draft genome for muskoxen, the only living member of the *Ovibos* genus. A combination of paired-end and in silico mate-pair libraries resulted in an assembly with an N50 of 13 Mb and a BUSCO score of 88.7%. The application of in silico mate pair libraries greatly improved genome scaffolding and genome quality but does not fully the replace the need for further sequencing of long read data, mate pair libraries, and/or sequencing by ligation to further enhance the description of the muskox genome. Beyond the development of a draft genome assembly and annotation, these data were used to reconstruct a phylogenetic tree, estimate the divergence time of this unique genus, and assess trends in historical effective population sizes via pairwise sequentially Markovian coalescent (PSMC) analyses. Divergence estimates were consistent with previous studies using mitochondrial DNA, while PSMC analyses found effective population lows coinciding with major glaciation events. With the addition of genomes from diverse muskox populations, future research should include the identification of positively selected genes to gain insight into the muskox’s key Arctic adaptations whose genetic underpinnings remain unknown. Overall, these data provide a solid foundation for further genome sequencing to elucidate patterns of gene flow, drift, and selection to better understand the muskox’s varying demographic and evolutionary histories and their capacity to adapt to rapid environmental change.

## Figures and Tables

**Table 1:** Comparison of quality metrics for genome assemblies pre- and post-cross-species-scaffolding and pre- and post-mitochondrial read filtering. Mx1 is the initial scaffold level assembly prior to both cross-species scaffolding and mitochondrial read filtering; Mx2 is the scaffold level genome assembly after cross-species scaffolding, but prior to mitochondrial DNA filtering; Mx3 is the scaffold level genome after mitochondrial read filtering, but prior to cross-species scaffolding; Muskox Final is the final genome assembly with columns for both contig and scaffold levels of assemblies.

Program	Quality Metric	Mx1	Mx2	Mx3	Muskox Final (Contig)	Muskox Final (Scaffold)
QUAST	Assembly length	2,528,575,139	2,704,466,437	2,527,322,807	2,473,404,411	2,621,890,883
	Assembly length over 25,000 bp	1,310,898,307	2,687,729,513	1,275,654,848	1,683,252,947	2,601,612,364
	Number of contigs	822,935	6495	868,473	114,189	8659
	Longest contig	328,372	9,453,317	301,459	388,735	50,595,910
	L50	27,014	494	26,422	18,651	61
	N50	26,107	1,705,149	26,274	38,369	13,200,690
	N per 100 kbp	5056.29	10,272	5035	0.93	5664
	GC content	41.56	41.81	41.55	44.53	44.53
BUSCO	Completed BUSCO (out of 3023)	1984 (65.6%)	2646 (87.6%)	1953 (64.6%)	N/A	2673 (88.4%)
	Single Copy Completed BUSCO (out of 3023)	1973 (65.2%)	2623 (86.7%)	1941 (64.2%)		2658 (87.9%)
	Duplicated BUSCO (out of 3023)	14 (0.4%)	14 (0.4%)	12(0.4%)		15 (0.5%)
	Fragmented (out of 3023)	448 (14.8%)	223 (7.1%)	458(15.2%		212 (7%)
	Missing (out of 3023)	591 (19.5%)	163 (5.3%)	612(20.2%)		138 (4.6%)

**Table 2:** Comparison of the final muskox genome assembly to both another Arctic ruminant (reindeer) and the highest quality wild ruminant genomes assembled by Chen et al. [45].

	*Ovibos moschatus*	*Okapia johnstoni*	*Rangifer tarandus*	*Gervus albirostris*	*Procapra przewalskii*	*Capra ibex*	*Ovis Amon*
Common name	Muskox	Okapi	Reindeer	White-lipped deer	Przewalski’s gazelle	Ibex	Argali
Scaffold N50	13,200,690	3,620,116	1,059,113	3,567,448	5,152,914	15,190,720	5,734,776
Contig N50	38,369	58,892	91,805	22,599	20,018	24.835	45,638
BUSCO %	88.4%	90.10%	90.40%	89.10%	89.10%	92.50%	93.10%
Number of genes	19,132	19,568	21,555	23,319	23,562	21,204	20,335
Average cds	1461	1518	1440	1440	1150	1544	1571

## Data Availability

The data sets supporting the results of this article are available on GenBank, under the BioProject (Accession: PRJNA640637).

## Supplementary Materials

The following supporting information can be downloaded at: https://www.mdpi.com/article/10.3390/genes13050809/s1, Figure S1: Nx and cumulative length plot of the muskox reference genome; Figure S2: Barplot of number of genes with Blast2Go output; Table S1: Library sequencing statistics for reference genome assembly; Table S2: Comparison of Genome assemblies.
