# Supplementary material for: Draft Genome Assembly of an Iconic Arctic Species: Muskox (Ovibos moschatus)
Source: Genes (Basel). 2022 May 1;13(5):809. doi: 10.3390/genes13050809 (PMC9140810; doi:10.3390/genes13050809)
Supplement: Supplementary file 1 [file genes-13-00809-s001.zip › Muskox Genome Supplemental/Genes-Muskox genome Supplementary Figures.pdf]

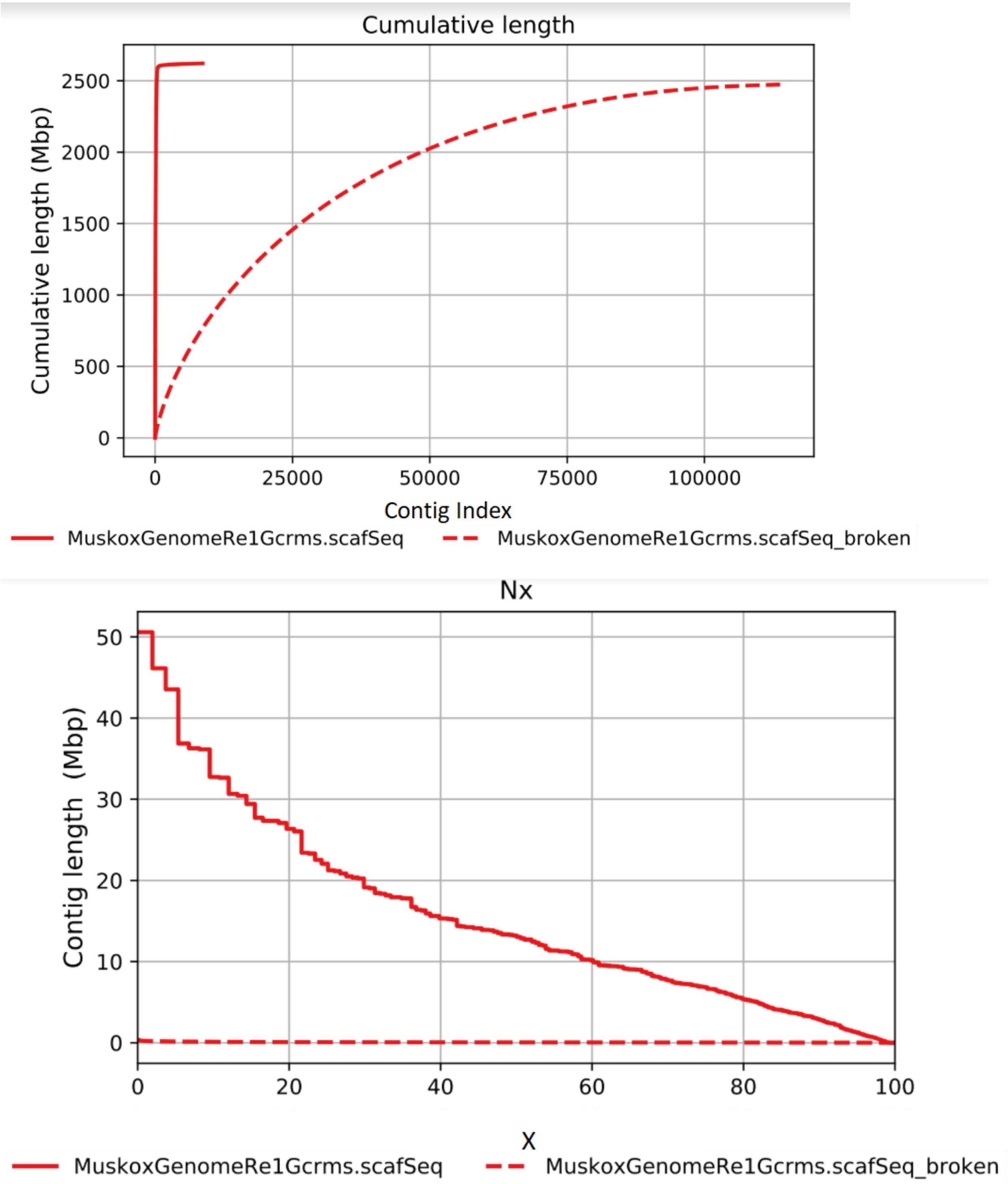

Figure S1. Nx and cumulative length plot of muskox reference genome assembly based on Quast analyses.

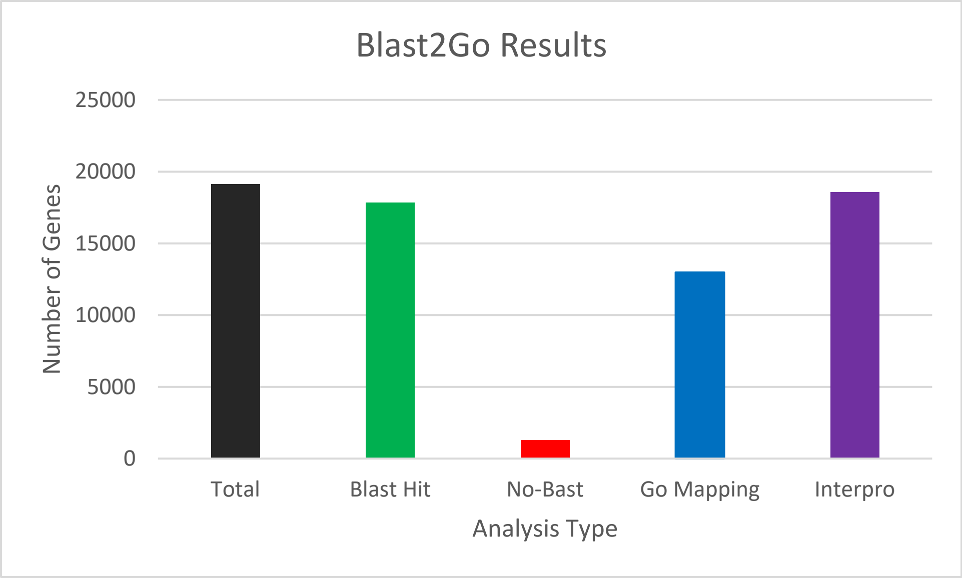

Figure S2. Barplot of number of genes with Blast2Go output
